# Fellowships, Grants, & Awards

**Published:** 2006-02

## Genetic and Genomic Analyses of *Xenopus*

For the past few years, the international research community has been generating genetic and genomic data and reagents for the model systems, *X. tropicalis* and *X. laevis*. These products include: 1) cDNA libraries and EST sequences; 2) UniGene clusters; 3) full-insert cDNA clones and sequences; 4) a genetic map; 5) genomic libraries and genomic sequences; 6) a physical map; 7) genome sequence; 8) micro-arrays, and 9) transgenic and mutant animals (see http://www.nih.gov/science/models/xenopus). These diverse data and reagents are being generated by investigators from several different research communities, including geneticists, gene sequencers, gene mappers, cell biologists, developmental biologists, and bioinformatics experts. These materials and data can now be used to enhance the role of *Xenopus* as a model system. This PAR solicits applications from investigators in these different fields to combine these data, reagents and methodologies to elucidate the genetic basis of cell biological events, including embryonic development and organogenesis.

The PAR solicits applications to utilize the newly generated methodologies and reagents, such as the clones and sequence information, to identify and characterize genes, gene families, and gene networks that control developmental and cellular events. It also solicits applications to generate research tools and to perform pilot studies. Additionally, it solicits applications to devise and improve techniques to alter gene expression and to control the spatial and temporal pattern of gene expression.

Examples of projects include:

- Larger projects from multidisciplinary teams that are based on extensive preliminary data. These applications could use the newly available genetic and genomic data and reagents, and/or new methodologies to characterize and study genes involved in developmental events and in cellular processes. These projects will be particularly appropriate for collaborations between investigators with different expertise to analyze the genomic data to elucidate the genetic bases of cellular and developmental events.
- Smaller projects using existing techniques to generate research tools, such as genetic and physical maps, and to mine data; and projects to perform pilot studies such as phenotypic screens, microarray analyses, and expression cloning designed to produce preliminary data that would form the basis of future applications.
- Smaller projects designed to develop novel techniques, such as strategies for mutagenesis, conditional gene expression, or for identifying new genes or mutants, as well as new tools to mine and analyze sequence data.

Objectives to be addressed in applications submitted in response to this PA include, but are not limited to, the following: 1) development and/or application of novel methods of mutagenesis (e.g., insertional, site-specific, conditional knockout vectors or systems); 2) development and/or use of techniques supporting more efficient targeting of induced local lesions in genomes (TILLING); 3) development and/or use of technologies for gene inactivation and for gene expression manipulation including, but not limited to, morpholino oligonucleotides, new types of antisense technology, techniques for homologous recombination, techniques for gene trapping, and strategies for directing gene misexpression, or other transgenic methodologies; 4) development of high throughput small molecule screens; 5) development of new genetic or genomic resources that are of high priority for the *Xenopus* community; 6) development and/or application of novel screens for mutants. These may be refinements of phenotypic analyses preparatory to screening, or phenotypic screens based on observation of alterations in development, morphology, or physiology; 7) screens focusing on identifying novel developmental genes and pathways.

This funding opportunity will use the modular NIH Individual Research Project Grant (R01) award mechanism. As an applicant, you will be solely responsible for planning, directing, and executing the proposed project.

This funding opportunity uses just-in-time concepts. It also uses the modular as well as the non-modular budget formats (see http://grants.nih.gov/grants/funding/modular/modular.htm). Specifically, if you are submitting an application with direct costs in each year of $250,000 or less, use the modular budget format described in the PHS 398 application instructions. Otherwise follow the instructions for nonmodular research grant applications.

The PHS 398 application instructions are available at http://grants.nih.gov/grants/funding/phs398/phs398.html in an interactive format. Applicants must use the currently approved version of the PHS 398. For further assistance contact GrantsInfo at 301-435-0714 (telecommunications for the hearing impaired: TTY 301-451-0088) or by e-mail: GrantsInfo@nih.gov.

Applications must be prepared using the current PHS 398 research grant application instructions and forms. Applications must have a D&B Data Universal Numbering System (DUNS) number as the universal identifier when applying for Federal grants or cooperative agreements. The D&B number can be obtained by calling 866-705-5711 or through the web site at http://www.dnb.com/us/. The D&B number should be entered on line 11 of the face page of the PHS 398 form.

The letters of intent receipt dates for this PAR are December 19, 2005, 2006, 2007, with the application receipt dates January 18, 2006, 2007, 2008. The complete version of this PAR is available at http://grants/guide/pa-files/PAR-05-166.

Contact: Steven L. Klein, Developmental Biology, Genetics and Teratology Branch, National Institute of Child Health and Human Development, Room 4B01, 6100 Executive Boulevard, Bethesda, MD 20892 USA, Rockville, MD 20852 USA (for express/courier service; non-USPS service), 301-435-6886, e-mail: kleins@mail.nih.gov. Reference PAR-05-166

## Mentored Research Scientist Development Award (K01)

The Mentored Research Scientist Development Award (K01) provides support for a sustained period of protected time for intensive research career development under the guidance of an experienced mentor, or sponsor, in the biomedical, behavioral, or clinical sciences leading to research independence. The expectation is that through this sustained period of research career development and training, awardees will launch independent research careers and become competitive for new research project grant (R01) funding.

Although all of the participating NIH Institutes and Centers use this support mechanism to support career development experiences that lead to research independence, some Institutes and Centers use this award for individuals who propose to train in a new field or for individuals who have had a hiatus in their research career because of illness or pressing family circumstances. Other Institutes and Centers utilize the K01 to increase research workforce diversity by providing enhanced research career development opportunities. Therefore, potential applicants are strongly advised to contact the appropriate NIH staff member identified in Section VII to discuss their particular circumstances before developing an application.

The candidate must devote a minimum of 75% of full-time professional effort to the goals of this award. The remainder may be devoted to clinical, teaching, or other research pursuits consistent with the objectives of the award. Both the didactic and research phases of an award period must be designed to develop the necessary knowledge and research skills in scientific areas relevant to the career goals of the candidate.

All career development programs must be tailored to meet the individual needs of the candidate. The candidate and mentor are jointly responsible for the preparation of the plan for the career development program. Applicants must justify the need for this award and provide a convincing case that the proposed period of support will substantially enhance their careers as independent investigators in their chosen area of research. The sponsoring institution must be able to demonstrate a commitment to the development of the candidate as a productive, independent investigator.

This funding opportunity will use the NIH K01 award mechanism. As an applicant, the candidate and his/her mentor will be jointly responsible for planning, directing, and executing the proposed project and career development activities.

This funding opportunity uses the just-in-time budget concepts. It also uses the nonmodular budget format described in the PHS 398 application instructions (see http://grants.nih.gov/grants/funding/phs398/phs398.html). The applicant should follow the PHS 398 instructions for budget information, providing only the total direct costs for each year and the entire proposed period of support and budget justification information.

The PHS 398 application instructions are available at http://grants.nih.gov/grants/funding/phs398/phs398.html in an interactive format. Applicants must use the currently approved version of the PHS 398. For further assistance contact Grants Info 301-435-0714 (telecommunications for the hearing impaired: TTY 301-451-0088) or by e-mail: GrantsInfo@nih.gov.

Applications must be prepared using the most current PHS 398 research grant application instructions and forms. Applications must have a Dun and Bradstreet (D&B) Data Universal Numbering System (DUNS) number as the universal identifier when applying for federal grants or cooperative agreements. The D&B number can be obtained by calling 866-705-5711 or through the web site at http://www.dnb.com/us/. The D&B number should be entered on line 11 of the face page of the PHS 398 form.

The application receipt dates for this PA are available at http://grants.nih.gov/grants/funding/submissionschedule.htm. The complete version of this PA is available at http://grants.nih.gov/grants/guide/pa-files/PA-06-001.

Contact: Applicants should refer to the NIH web site (http://grants.nih.gov/grants/guide/contacts/pa-06-001_contacts.htm) for information regarding each IC's scientific/research contacts for this K01 program. Reference PA-06-001

## Mentored Quantitative Research Development Award (K25)

A particular area of research is often invigorated by novel perspectives that may be provided by individuals trained outside that research arena. In an effort to advance research relevant to the mission of the National Institutes of Health (NIH) which includes basic biomedical, clinical biomedical, bioengineering, bioimaging, and behavioral research, the participating Institutes and Centers solicit applications for the Mentored Quantitative Research Career Development Award (K25). The K25 mechanism is meant to attract to NIH-relevant research those investigators whose quantitative science and engineering research has thus far not been focused primarily on questions of health and disease. Examples of quantitative scientific and technical backgrounds considered appropriate for this award include, but are not limited to, mathematics, statistics, economics, computer science, imaging science, informatics, physics, chemistry, and engineering. Potential applicants are strongly advised to contact the appropriate NIH staff member identified in Section VII (http://grants.nih.gov/grants/guide/contacts/pa-06-087_contacts.htm) to discuss their particular circumstances and quantitative backgrounds before developing an application.

The K25 Award will support the career development of quantitatively trained investigators who make a commitment to basic or clinical biomedicine, bioengineering, bioimaging, or behavioral research that is relevant to the NIH mission. This award provides support for a period of supervised study and research for productive professionals with quantitative backgrounds who have the potential to integrate their quantitative expertise with NIH-relevant research and develop into productive investigators. The K25 program is intended for research-oriented investigators from the postdoctoral level to the level of senior faculty.

The NIH is especially interested in increasing the number of scientists trained to conduct high-quality research that combines insights derived from, and cuts across, different scientific, technical, and biomedical areas. Accordingly, the Mentored Quantitative Research Career Development Award forms an important part of an initiative to attract talented individuals with highly developed quantitative skills to the challenges of research relevant to the mission of NIH. This initiative is consistent with the recommendations of the Bioengineering Education and Training Panel which was convened as part of the 1998 Bioengineering Consortium (BECON) Symposium (the symposium report is available at http://www.becon.nih.gov/becon_symposia.htm).

The Mentored Quantitative Research Career Development Award is intended to increase the availability of high-quality, multidisciplinary, didactic training, and research project guidance in the context of a mentored research career transition experience. Candidates interested in cross-disciplinary research will become well grounded in behavioral, biomedical, bioimaging, or bioengineering research. At the completion of the award, candidates should have both the knowledge and the skills necessary to compete for independent research support from NIH, or to participate as leading members of multidisciplinary research teams.

The specific objectives of the Mentored Quantitative Research Career Development Award (K25) are: 1) to encourage research-oriented quantitative scientists and engineers with little or no experience in biomedicine, bioengineering, bioimaging, or behavioral research to gain fundamental knowledge in these areas and develop relevant research skills, and to gain experience in current concepts, advanced methods, and experimental approaches that will allow them to conduct basic or clinical biomedical, behavioral, bioimaging, or bioengineering research, and to become independent investigators or play leading roles in multidisciplinary research teams; 2) to increase the pool of quantitative researchers who can conduct biomedical, behavioral, or bioengineering studies, capitalizing on the quantitative backgrounds of these investigators to inform new directions in biomedical, behavior and bioengineering research; 3) and to provide a unique opportunity for candidates holding degrees in quantitative science or engineering to embark on three to five years of special study, including course work, seminars, meetings, and mentored research, to achieve the career enhancement goals outlined above.

Because of the focus on a progression toward independence as a quantitative biomedical, behavioral, bioimaging, or bioengineering researcher, the prospective candidate for the Mentored Quantitative Research Career Development Award will require enhanced skills in the experimental, theoretical, and conceptual approaches used in biomedicine, behavioral science, bioimaging, or bioengineering. To satisfy this requirement, the candidate should propose a period of study and career development that is complementary to his or her previous research and experience. For example, a candidate with no or very limited experience in a given field of biomedical research may find a phased developmental program lasting for five years that includes a designated period of didactic training together with a closely supervised research experience the most efficient means of attaining independence. A candidate with, for example, more research experience in biomedicine may benefit from a program with greater emphasis on appropriate laboratory research with lower levels of supervision and direction. All programs should be carefully tailored to meet the individual needs of the candidate and must include (an) active mentor(s) who is (are) competent and willing to provide the appropriate research guidance.

Candidates should strongly consider incorporating into their training plan formal courses in relevant areas of biomedicine, behavioral science, bioimaging, or bioengineering; this program offers a unique opportunity to devote protected time to this activity.

The candidate must devote a minimum of 75% of full-time professional effort to the goals of this award. The remainder may be devoted to teaching or other research pursuits consistent with the objectives of the award. Both the didactic and research phases of an award period must be designed to develop the necessary knowledge and research skills in scientific areas relevant to the career goals of the candidate.

All research career development programs must be tailored to meet the individual needs of the candidate. The candidate and mentor are jointly responsible for the preparation of the plan for the career development program. Applicants must justify the need for this award and provide a convincing case that the proposed period of support will substantially enhance their careers as independent investigators in their chosen area of quantitative research. The sponsoring institution must demonstrate a commitment to the development of the candidate as a productive, independent investigator.

This funding opportunity will use the NIH K25 award mechanism. As an applicant, the candidate and his/her mentor will be jointly responsible for planning, directing, and executing the proposed project and career development activities.

This funding opportunity uses the just-in-time budget concepts. It also uses the nonmodular budget format described in the PHS 398 application instructions (see http://grants.nih.gov/grants/funding/phs398/phs398.html). The applicant should follow the PHS 398 instructions for budget information, providing only the total direct costs for each year and the entire proposed period of support and budget justification information.

The K25 project period may be for up to five years, with a minimum of three years required. Awards are not renewable and are not transferable from one principal investigator to another.

The PHS 398 application instructions are available at http://grants.nih.gov/grants/funding/phs398/phs398.html in an interactive format. Applicants must use the currently approved version of the PHS 398. For further assistance contact Grants Info, 301-435-0714 (telecommunications for the hearing impaired: TTY 301-451-0088), e-mail: GrantsInfo@nih.gov.

Applications must be prepared using the most current PHS 398 research grant application instructions and forms. Applications must have a Dun and Bradstreet (D&B) Data Universal Numbering System (DUNS) number as the universal identifier when applying for federal grants or cooperative agreements. The D&B number can be obtained by calling 866-705-5711 or through the web site at http://www.dnb.com/us/. The D&B number should be entered on line 11 of the face page of the PHS 398 form.

The application receipt dates for this PA are available at http://grants.nih.gov/grants/funding/submissionschedule.htm. The complete version of this PA is available at http://grants.nih.gov/grants/guide/pa-files/PA-06-087.

Contact: Applicants should refer to the NIH web site (http://grants.nih.gov/grants/guide/contacts/pa-06-087_contacts.htm) for information regarding each IC’s scientific/research contacts for this K25 program. Reference PA-06-087

